# Case Report: Rare multisystem metastasis in head and neck paraganglioma with *SDHB* pathogenic variant and *KIF1B* VUS manifested as FUO

**DOI:** 10.3389/fendo.2025.1612259

**Published:** 2025-08-21

**Authors:** Yongshi Yang, Xiaotong Tian, Congwei Jia, Hongwei Fan, Taisheng Li, Li Zhang, Zhengyin Liu

**Affiliations:** ^1^ Department of Allergy, Peking Union Medical College Hospital, Chinese Academy of Medical Science and Peking Union Medical College, Beijing, China; ^2^ Department of Infectious Disease, Peking Union Medical College Hospital, Chinese Academy of Medical Sciences and Peking Union Medical College, Beijing, China; ^3^ Department of Pathology, Peking Union Medical College Hospital, Chinese Academy of Medical Sciences and Peking Union Medical College, Beijing, China

**Keywords:** paraganglioma, metastasis, *SDHB*, case report, gene variant, FUO, KIF1B

## Abstract

**Background:**

Paragangliomas (PGLs) are rare neuroendocrine tumors originating from the extra-adrenal autonomic paraganglia with a strong genetic background. *SDHB* pathogenic variants are associated with the highest rate of malignancy in PGLs. Most head and neck paragangliomas (HNPGs) are asymptomatic and benign, and multiple metastases are rare.

**Case presentation:**

A 37-year-old man presented at our hospital with a fever of unknown origin (FUO) without any other complaints except for mild consumption lasting over 6 months. Laboratory examinations showed elevated inflammatory markers (CRP, ESR, IL-6, and Ferritin) but no abnormalities in infection, immune, or tumor markers. Imaging examination found an oval-shaped space-occupying lesion in the right parapharyngeal space, with rare and unique vertebral imaging findings. Octreotide imaging and 68Ga-FAPI PET/CT scans indicated a potential for neuroendocrine tumors with lymph nodes, bone, and lung metastases. Pathology demonstrated metastatic paraganglioma. Whole-genome sequencing identified an *SDHB* pathogenic variant and a *KIF1B* variant of uncertain significance. Following multidisciplinary consultation, the patient opted for the cyclophosphamide-vincristine-dacarbazine (CVD) chemotherapy regimen and was subsequently transferred to a regional hospital for coordinated follow-up.

**Conclusions:**

This case reported rare co-occurring variants in *SDHB* and *KIF1B* and unusual imaging findings of metastasis in paraganglioma. A multimodal imaging evaluation and whole-genome sequencing were instrumental in assessing paraganglioma patients. This case suggested that atypical imaging features should raise suspicion of malignant diseases and underscored the importance of interdisciplinary collaboration in guiding the diagnosis and treatment of complex and rare clinical cases.

## Introduction

Paragangliomas (PGLs) are rare neuroendocrine tumors originating from the extra-adrenal autonomic paraganglia (1). Previous studies have shown that the incidence rates of pheochromocytoma (PCC) and sympathetic paraganglioma (sPGL) have increased significantly over the past two decades, with about 0.6 cases per 100,000 person-years (2). Clinically, the sPGL are characterized by catecholamine hypersecretion and predominantly localize to the sympathetic paravertebral ganglia of the thorax, abdomen, and pelvis. In contrast, most parasympathetic PGLs are nonfunctional, and the majority of parasympathetic PGLs arise in the head and neck (3).

With the development of next-generation sequencing, approximately 30%-50% of PGLs are associated with hereditary syndromes (4). To date, more than 20 susceptibility genes have been found to be associated with the development of hereditary PCC and PGL (PPGLs), such as *VHL*, *SDHB*, *SDHD*, *SDHA*, *SDHC*, *NF1*, and *RET*, which are considered the most common pathogenic genes. Other susceptibility genes, which have not yet undergone rigorous genotype–clinical outcome evaluation, include *KIF1B*, *IDH1*, *HIF2A*, *MDH2*, *FH*, *SLC25A11*, and *DNMT3A* (3, 5). Some hereditary paragangliomas, particularly head and neck PGLs (HNPGLs), are predominantly associated with the pseudohypoxia cluster and germline succinate dehydrogenase subunit *SDHD* and *SDHB* variants, indicating the need for genetic counseling and testing for all patients with PGLs (6). Many studies have shown that *SDHB* gene pathogenic variants are associated with the highest risk of malignancy and represent the most frequent cause of metastatic paragangliomas (7, 8). We herein present a diagnostically challenging case of fever of unknown origin (FUO), which was ultimately diagnosed with HNPGLs harboring an *SDHB* pathogenic variant and a *KIF1B* variant of uncertain significance (VUS), complicated by extensive bone and pulmonary metastases.

## Case presentation

A 37-year-old man presented at our hospital with recurrent fever for over 6 months, without any other discomforts. Since March 2024, he had developed an intermittent fever in the afternoon, with a temperature max (Tmax) of 38.3°C, which could gradually return to normal without medication. The patient visited the local hospital, where laboratory tests showed a significant increase in inflammatory markers, including C-reactive protein (CRP) 154.5mg/L and erythrocyte sedimentation rate (ESR) 100mm/h, accompanied by mild anemia. A Computed Tomography (CT)-guided percutaneous puncture biopsy of the lumbar vertebral space-occupying lesions was performed, but pathology showed no clear evidence of malignant lesions. The local hospital was unable to provide a definitive diagnosis and attempted intermittent treatment with prednisone and broad-spectrum antibiotics therapy. However, the patient still had recurrent fevers.

Consequently, the patient was admitted to our hospital for investigation of FUO. Physical examination showed that the patient was emaciated, with a body mass index (BMI) of 15.78kg/m^2^, presenting an anemic appearance. There was no swelling of superficial lymph nodes. The spine showed no deformity and tenderness, and no obvious tenderness or percussion pain in the spinous process and paravertebral region. Limb activity was good, and limb muscle strength was normal. The patient has had anemia since childhood. He has a long-term smoking and drinking history. The patient reported no history of cancer or endocrine disorders in his parents, one older brother, one younger sister, and his son.

Laboratory tests showed the CRP 178.34mg/L, ESR 81mm/h, IL-6 42.2pg/mL, and Ferritin 753ng/mL, all significantly increased. Bone marrow culture, bone marrow tissue pathogen metagenomic next-generation sequencing (mNGS), as well as screening for Parasites, Brucella, Tuberculosis, and other infection-related indicators, and no suspected pathogenic pathogens were detected. The autoimmune antibodies spectrum, anti-neutrophil cytoplasmic antibodies, immunoglobulin, serum IgG subclasses, complement, and other immune disease-related indicators were all negative. There were no significant abnormal results in the examination of tumor-related indicators such as tumor serum markers, serum/urine protein electrophoresis, serum/urine immunofixation electrophoresis, angiotensin-converting enzyme (ACE), and the vascular endothelial growth factor (VEGF).

**Figure 1:**
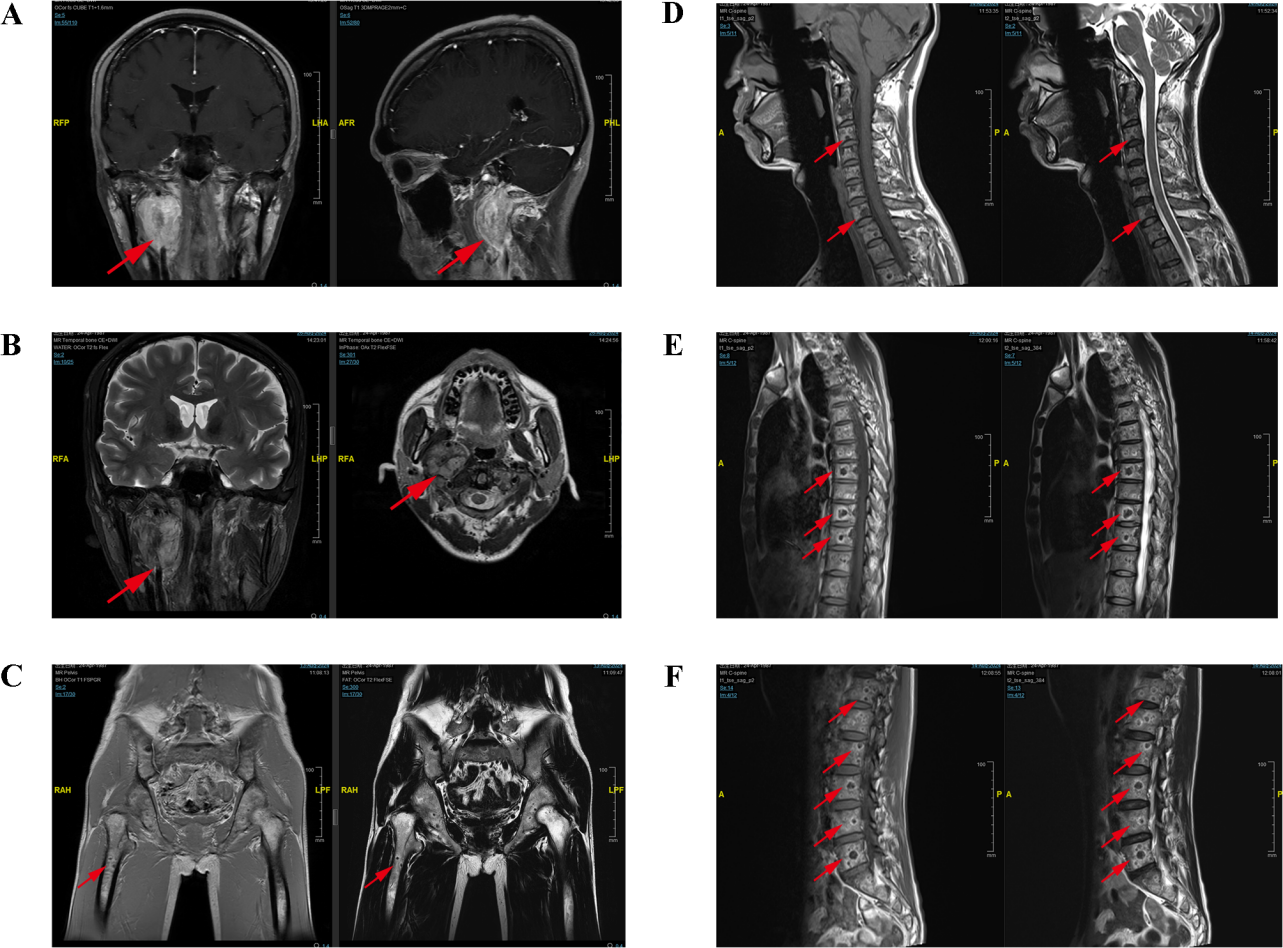
The MRI examination images of the patient. The MRI of the head **(A, B)** demonstrated an oval-shaped space-occupying lesion between the internal carotid artery and vein in the right parapharyngeal space, exhibiting uneven signal intensity and heterogeneous enhancement, with a larger section measured approximately 38mm×29mm×43mm. The MRI of the pelvis **(C)**, cervical vertebra **(D)**, thoracic vertebra **(E)** and lumbar vertebra **(F)** indicated the presence of multiple flaky and quasi-circular abnormal signal shadows in the pelvic bones and vertebral bodies. The MRI showed multiple patchy, quasi-circular abnormal signal shadows, with low signal intensity on T1WI and T2WI accompanied by surrounding ring-shaped high signal intensity shadows and slightly high signal intensity on fat-suppressed T2WI accompanied by peripheral annular low signal intensity shadows, which showed special ring signs.

**Figure 2:**
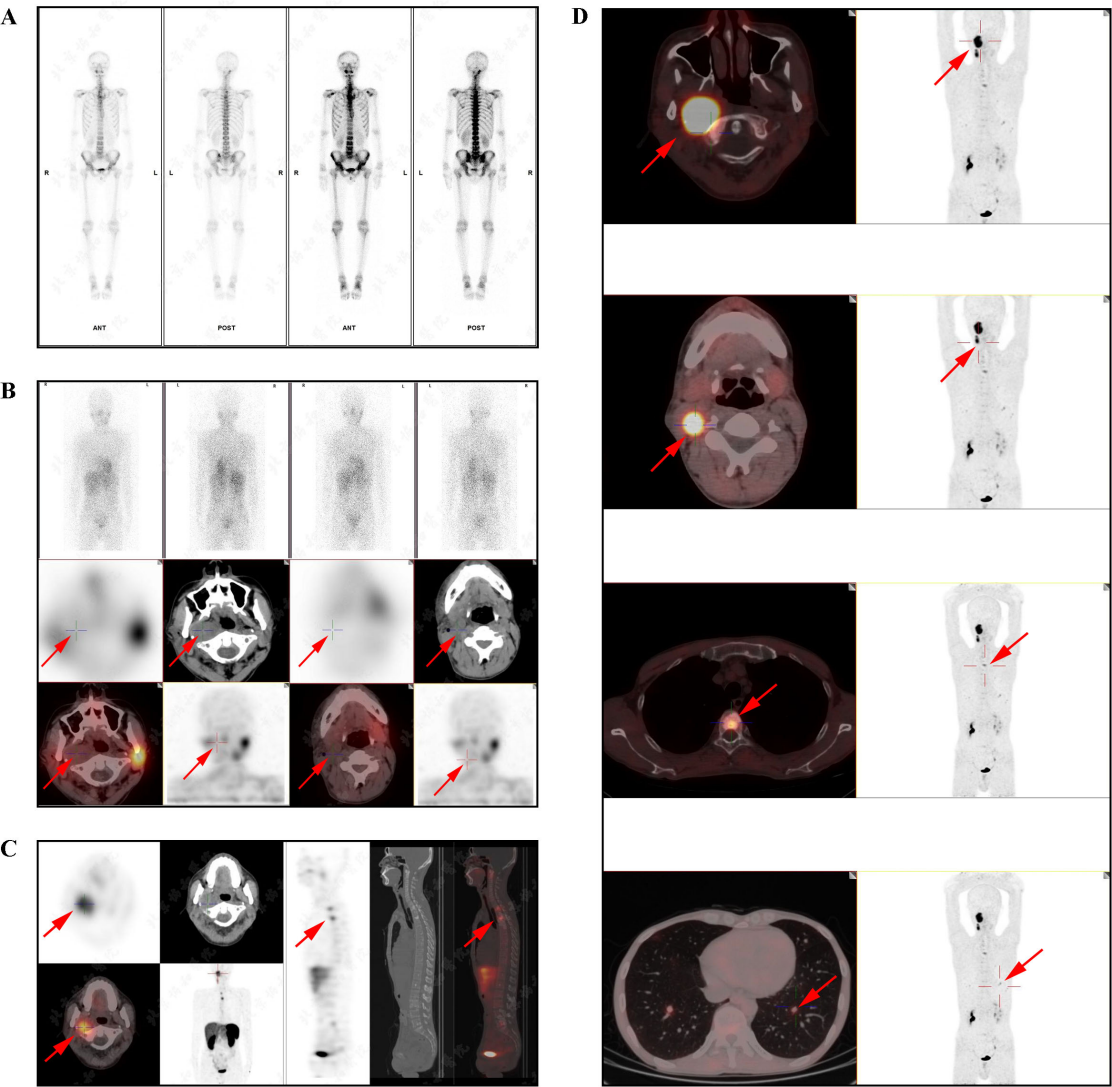
The nuclear medicine examination images of the patient. **(A)** The whole-body bone imaging indicated an abnormal increase in radioactive uptake in the right skull base bone and uneven distribution of radioactivity in some cervical and thoracic vertebrae. **(B)** SPECT/CT fusion imaging (MIBG): swollen lymph nodes in the right parapharyngeal space and right region II, multiple high-density nodules in the cervical and thoracic vertebrae, and the left second posterior rib, without radioactive uptake. **(C)** SPECT/CT fusion imaging (Octreotide): a tumor with heightened somatostatin receptor expression was observed in the right parapharyngeal space, measuring approximately 2.8×2.3cm; multiple high-density shadows were detected in the spine, pelvis, and left clavicle, bilateral scapulae, left humerus, and left femur, particularly prominent in the spine, among which larger lesions (T1 and T3 vertebral lesions) showed significantly increased radioactive uptake. **(D)** The PET/CT scans revealed a tumor in the right parapharyngeal space with heightened FAPI expression, measuring approximately 4.1×2.7cm in size and exhibiting a maximum standardized uptake value (SUVmax) of 7.9. Several lymph nodes in the deep cervical region (zone II) showed increased expression of somatostatin receptors. Diffuse multiple high-density shadows were observed in the cervical, thoracic, and lumbar vertebrae, and the left iliac bone, with some areas showing heightened FAPI expression (SUVmax of 3.8). Multiple micro and small nodules were detected in both lungs, with the larger one located in the lower lobe of the left lung, measuring approximately 1.0cm in diameter, with heightened FAPI expression (SUVmax of 2.7).

Contrast-enhanced CT revealed multifocal micro- and small pulmonary nodules bilaterally, scattered vertebral body density abnormalities, and a right parapharyngeal space mass with heterogeneous prominent enhancement (CT value 65–118 HU), suspicious for paraganglioma. **Figure 1** shows the patient’s MRI images, including head (A&B), pelvis (C), cervical (D), thoracic (E), and lumbar (F) vertebrae. These scans, with imaging features consistent with paraganglioma, further confirmed the diagnosis and suggested multiple metastases. Whole-body bone scintigraphy (**Figure 2A**) revealed cervical and thoracic vertebral abnormalities suggesting malignancy. Meta-iodobenzylguanidine (MIBG) scintigraphy (**Figure 2B**) showed no abnormal tracer uptake in adrenal regions or elsewhere. Octreotide imaging (**Figure 2C**) demonstrated somatostatin receptor overexpression in the right parapharyngeal tumor, spine, and bilateral lung nodules, indicating metastatic disease. 68Ga-FAPI PET/CT (**Figure 2D**) confirmed a malignant right parapharyngeal tumor and revealed FAPI-expressing metastases in cervical lymph nodes, spine, left ilium, and bilateral lungs. Based on the imaging findings, we performed additional bone metabolism and endocrine function tests. Results revealed no significant abnormalities in these parameters (**Supplementary Table S1**).

**Figure 3:**
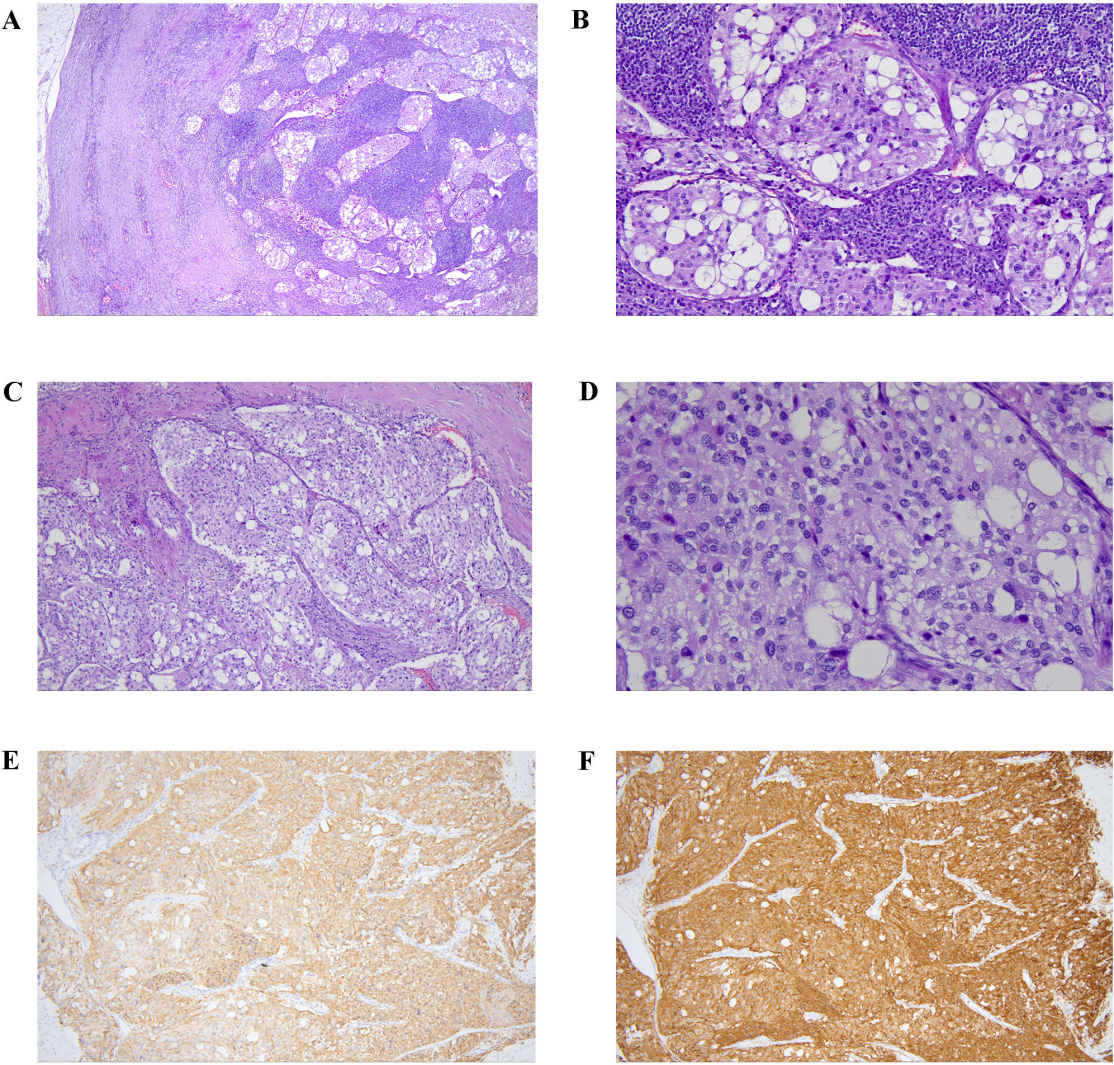
The pathological of the tumor tissue and the lymph node tissue. Tumor metastasis was observed in the sinus of lymph node (**A**, low power, HE ×40; **B**, medium power, HE ×200). Tumor cells were arranged in nest bulk pattern with abundant collagenous stroma (**C**, medium power, HE ×100), and had abundant pale cytoplasm and hyperchromatic nuclei (**D**, high power, HE ×400). Immunostaining showed tumor cells express chromogranin A (**E**, medium power, ×100) and synaptophysin (**F**, medium power, ×100).

Before the biopsy, a comprehensive perioperative risk assessment was conducted to exclude major surgical contraindications, including infection, uncontrolled hypertension or hyperglycemia, hematological/coagulation abnormalities, anatomical risks, and anesthetic fitness. Preoperative evaluation was coordinated by multidisciplinary teams including otolaryngology, general surgery, interventional radiology, and anesthesiology. Finally, the otolaryngology-head and neck surgery team performed a lateral neck approach under general anesthesia for skull base tumor biopsy. No procedure-related adverse events were observed. The lymph nodes in zone II were enlarged and adhered. The lower pole of the tumor was located deep at the bifurcation of the internal and external carotid arteries, with a rich blood supply and hard texture. Part of the tumor was resected and sent for frozen section examination and pathological analysis. The pathological results are as follows: in the fibrous tissue of the tumor in the right parapharyngeal space, highly compressed dark-stained cells were observed. Immunohistochemical results (**Figure 3**) showed: AE1/AE3(-), CD20(-), CD3(-), Ki-67(index 5%), INSM-1(+), CgA(+), Syn(+); findings were consistent with neuroendocrine tumor. Tumor cells were observed in the lymph node tissue of the right II region. The immunohistochemical results were AE1/AE3(-), GATA3(-), INSM-1(+), CgA(+), Ki-67(index 5%), S-100(local scattered +), Syn(+), MGMT(partial +), SDHB(+), PIT-1(-), T-PIT(-), SF-1(-); EBER ISH(-); the diagnosis was considered as a metastatic neuroendocrine tumor, with a tendency towards paraganglioma.

**Figure 4:**
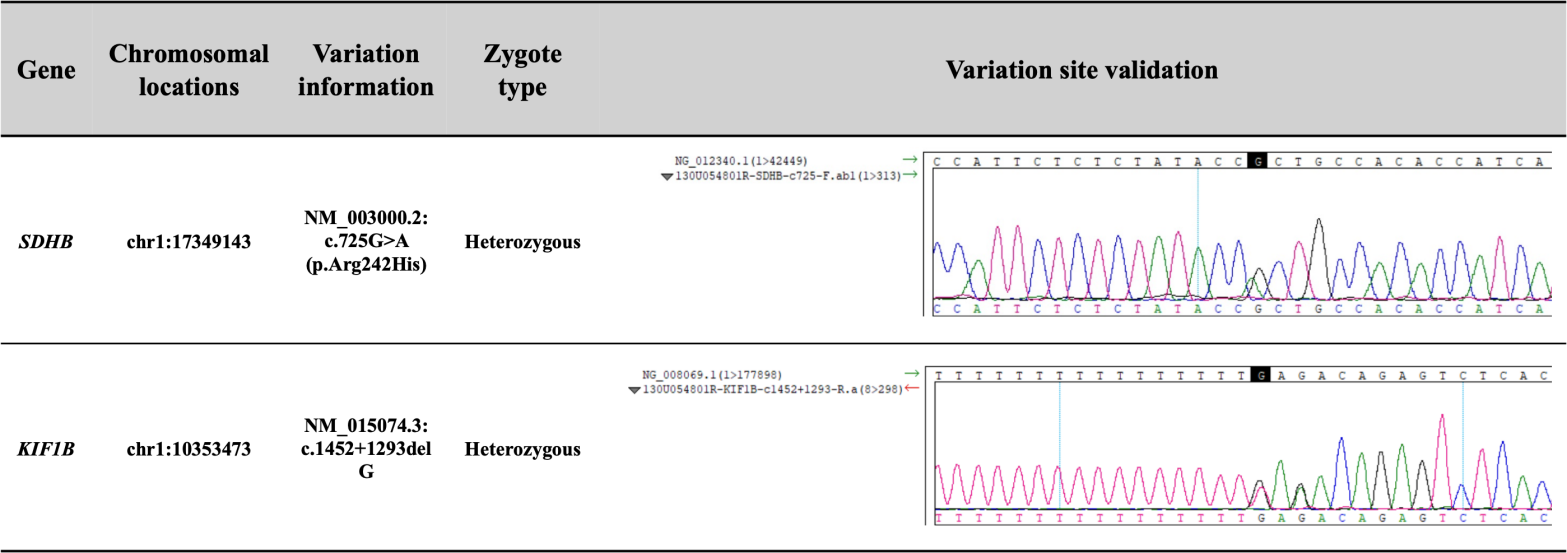
The results of the whole-genome sequencing on the genomic DNA from the patient’s peripheral blood. According to ACMG guidelines, the *SDHB* variant was referred to as pathogenic, supported by evidence codes: PP4 + PS3 + PP3 + PS4_Moderate + PM2. The *KIF1B* variant was classified as a variant of uncertain significance based solely on PP3.

Additionally, we performed whole-genome sequencing on the genomic DNA extracted from the patient’s peripheral blood. Molecular methods and bioinformatic analyses of whole-genome sequencing are summarized in the **Supplementary Materials**. The results (**Figure 4**) showed that the patient carried an *SDHB* gene substitution variant (NM_003000.2: c.725G>A; p.Arg242His), and a *KIF1B* gene deletion variant (NM_015074.3: c.1452 + 1293delG). Both variants were heterozygous. The chromosomal locations of these variants were at chr1:17349143 and chr1:10353473, respectively. According to ACMG (American College of Medical Genetics and Genomics) guidelines, the *SDHB* variant was referred to as pathogenic, while the *KIF1B* variant was classified as a VUS.

Finally, after evaluation by the oncology department, it was recommended to choose anti-VEGFR TKI therapy or peptide receptor radionuclide therapy (PRRT), including Sunitinib or Cabozantinib, or CVD regimen (cyclophosphamide, vincristine, and dacarbazine) treatment. The patient ultimately chose the CVD regimen and returned to the local hospital for further treatment.

## Discussion

Head and neck PGLs (HNPGLs) are extremely rare, comprising 3% of all PGLs and accounting for only 0.5% of all tumors in this region, which are very slow-growing and are often undetected for several years (9). Although most HNPGLs are benign, only 6% to 19% of HNPGLs may metastasize, which seriously affects survival (10). A review including 59 cases of HNPGLs extracted from the National Cancer Data Base showed five-year survival rates of 76.8% in patients with metastasis to regional lymph nodes and 11.8% in patients with distant metastasis (11). In this paper, we present a case that initially presented with FUO, and was ultimately diagnosed with HNPGLs with multiple metastases, accompanied by special imaging findings of bone metastasis, an *SDHB* pathogenic variant, and a *KIF1B* variant of uncertain significance.

The FUO is a challenging clinical syndrome with potential etiologies including infectious diseases, non-infectious inflammatory diseases, and neoplastic diseases, among others (12). It encompasses a broad differential diagnostic spectrum, and the causes exhibit significant geographical variations (13). In clinical practice, patients with PGLs with FUO as the main clinical manifestation are quite rare. Narechania S et al. reported a young healthy postpartum mother who presented with FUO and finally was found to have a retroperitoneal paraganglioma with metastases to the lungs and spine after appropriate diagnostic testing. And the genetic testing also identified a pathogenic variant in *SDHB* (14). Davies LL et al. also reported a patient with FUO and loin pain with elevated inflammatory markers and finally diagnosed with retroperitoneal paraganglioma confirmed by histological examination (15). Wang C et al. reported a 65-year-old man with retroperitoneal paraganglioma accompanied by elevated IL-6 levels and *KIF1B* single gene variant and without elevated levels of catecholamines and their derivatives, whose main symptoms were only fever and palpitations (16). Our patient was similar to this one with the IL-6 level increase and *KIF1B* gene variant. We also did not detect an increase in catecholamines and their metabolites in serum and urine. In addition, some studies have proposed that IL-6 secreting PCC should be considered in the differential diagnosis of FUO (17, 18). It is postulated that the IL-6 generated by the PCC is the culprit behind the fevers since the removal of the tumor, IL-6 levels declined, and the associated symptoms resolved (19, 20). However, it must be pointed out that compared to PCC, the relationship between IL-6 and PGLs still needs further confirmation.

PGLs are rare neuroendocrine tumors characterized by a strong genetic determinism. A review described the evolution of the genetics of paragangliomas and pheochromocytomas (PPGLs) over the past 20 years (5). According to the whole genome molecular genetic testing, this patient had rare double gene variants of *SDHB* and *KIF1B*. *SDHB* is a mitochondrial gene that is related to the metabolic activities of cells and tissues. It has attracted more and more attention from researchers due to its important structural functions affecting the occurrence and progress of related diseases (21). Compared with other hereditary subtypes of PPGLs, PPGLs with *SDHB* have the highest rates of disease-specific morbidity and mortality. PPGLs with *SDHB* are usually less differentiated and do not produce large amounts of catecholamines, which allows these tumors to grow sub-clinically for a long time (22). Some scholars believe that germline variants in the *SDHB* gene predispose to metastatic PGLs (5, 23). A meta-analysis found that the *SDHB* mutation had the highest prevalence in PPGLs (23%-31%), similar to the highest risk of metastasis posed by *SDHB* mutation (12%-41%) (24). The *KIF1B* gene is related to the protein called kinesin family member 1B, which is essential for the transport of materials within cells (25). However, research on the *KIF1B* gene in PPGLs is far less abundant than reports on other gene variants. In the study of Evenepoel L, somatic variants were identified in 54% of the PPGLs patients, and the *KIF1B* (20.4%) and *NF1* (20.8%) were the most frequently mutated genes (26). In 2020, a single-center study from our hospital included 314 PPGLs patients and found that 22.0% of patients had metastatic, 49.7% of patients were HNPGLs, and 107 variants were in 314 cases (34.1%). *SDHB* gene was the most frequently mutated gene (14.6%), followed by *RET* (3.8%), *VHL* (3.8%), *SDHD* (2.5%), *SDHA* (2.2%), *MAX* (1.9%), *FH* (0.3%), and *KIF1B* (0.3%) (27). Although some literatures have reported the identification of *KIF1B* gene variant in PPGLs patients, compared with *SDHB*, which is classified as a pathogenic variant gene, *KIF1B* gene variant can only be categorized as VUS. Further rigorous functional studies are still needed to confirm the clinical relevance. Over the last 20 years, significant progress has been made in the research of molecular biology and markers related to the aggressive behavior of PPGLs, but currently, there are still no clinical, biochemical, histopathological, or molecular markers that can be used to accurately predict the metastatic behavior of PPGLs at diagnosis (28).

It is reported that about 5-10% of cancer patients develop spinal metastasis during the disease process (29). The spine is the third most common site of metastatic disease, only following the lung and liver, and is also the most common site of bone metastasis (30). A retrospective study from our hospital included 250 patients with metastatic PPGLs and found that 85.6% had multisystem metastasis, with bone metastases being the most common site of metastasis (60.8%), following the lung (44.4%), lymph nodes (44.4%), and liver (30.8%). The incidence of germline variants, especially *SDHB* variants, was high in metastatic PPGLs patients, accounting for 76.5% (31). Using a large institutional database, Ayala-Ramirez M et al. conducted a retrospective study of 128 patients with pheochromocytoma or sympathetic paraganglioma. They found that 20% of the patients had metastatic disease located exclusively in the skeleton. The spine was the most common location of bone metastasis (81%), following the sacrum and pelvis (67%), proximal and distal long bones (49%), and skull (21%) (32). Further investigation is required to elucidate the roles of the *SDHB* pathogenic variant and the *KIF1B* variant of uncertain significance in driving the development of multiple metastases in this patient.

Another case report described a woman with *SDHB* variant and spinal metastatic PGL, presenting with multilevel lytic lesions at C1, C4-C7, L1-L3 (cervical/thoracic/lumbar spine) and compression fractures at C3 and T9 (33). Although they all have *SDHB* variant and extensive metastatic disease of the spine, this patient was significantly different from our reported patient in imaging and symptoms. In our report, the patient had no deformity of the spine, no tenderness, or percussion pain in the spinous process and paravertebral region, which seemed inconsistent with extensive imaging findings of vertebral metastasis. The patient’s vertebral MRI showed multiple patchy, quasi-circular abnormal signal shadows, with low signal intensity on T1WI and T2WI accompanied by surrounding ring-shaped high signal intensity shadows and slightly high signal intensity on fat-suppressed T2WI accompanied by peripheral annular low signal intensity shadows, which showed special ring signs. This was very similar to that reported by Mediouni A and his colleagues. They considered that the MRI of malignant HNPGLs had two types of lesions, namely nodular and expanding, and the nodular lesions showed specific imaging appearances, which were composed of low-intensity signals in the center, surrounded by a single fat-like halo or double halo (34). As reported by Yu and his colleagues, ring signs can be considered relatively specific signs in some other bone tumors, such as osteochondromyxoma in the Carney complex. They found the center of the ring signs had low or slightly equal signal intensities (compared with the muscle) on the T1WI and high or low signal intensities on the T2WI (35). The imaging findings of this patient also well remind us that when we find some special types of imaging appearance, we need to be alert to the possibility of malignant diseases.

The imaging localization examination of PGLs is beneficial for determining the selection of effective treatment methods. This patient underwent a very detailed imaging evaluation. Before surgery, temporal bone enhanced CT and MRI, as well as cervical CTA, were performed to clearly display the morphology, blood supply, and relationship with surrounding tissues of the tumor. The expert consensus recommends that PPGL patients with metastasis or inoperable conditions undergo ^131^I-MIBG nuclear imaging first and evaluate the possibility of ^131^I-MIBG treatment based on the functional and anatomical localization of the tumor (22). However, the ^131^I-MIBG radionuclide imaging of this patient was negative, so ^131^I-MIBG cannot be used for treatment. MIBG has low sensitivity in detecting metastatic, recurrent, located in the skull base, neck, chest, and bladder PGL, as well as PPGL genes associated with various subtypes of *SDHx* (especially *SDHB*); And the imaging effect is poor for low secretion function and small tumors, and false negatives may occur (36, 37). Somatostatin octreotide imaging (e.g., ^99m^Tc HTOC) can complement the ^131^I-MIBG imaging of PPGL and assist in diagnosis (38).

In summary, this case illustrated the detailed process of a fever of unknown origin and ultimately being diagnosed with paraganglioma and presented the rare imaging findings of vertebral metastasis in paraganglioma. This case highlighted the emphasis on interdisciplinary collaboration, including Otorhinolaryngology, Orthopedics, Oncology, Endocrinology, Pathology, Genetics, Anesthesiology, Nuclear Medicine, and Radiology, which formed a multidisciplinary team (MDT) to integrate clinical, imaging, pathological, and genetic insights for the comprehensive management of complex and rare clinical cases. Notably, this case identified rare co-occurring variants, an *SDHB* pathogenic variant and a *KIF1B* VUS, underscoring the critical need for comprehensive germline and somatic genetic testing in such cases, offering valuable insights and data for the diagnosis, management, and research of paraganglioma.

## Data Availability

The original contributions presented in the study are included in the article/**Supplementary Material**, and further inquiries can be directed to the corresponding authors.
