# Supplementary material for: Case Report: Rare multisystem metastasis in head and neck paraganglioma with SDHB pathogenic variant and KIF1B VUS manifested as FUO
Source: Front Endocrinol (Lausanne). 2025 Aug 21;16:1612259. doi: 10.3389/fendo.2025.1612259 (PMC12408261; doi:10.3389/fendo.2025.1612259)
Supplement: Supplementary file 1 [file Table1.docx]

**Supplementary Materials**

- **Molecular Methods and Bioinformatic Analyses of Whole-Genome Sequencing**

The Shenzhen BGI Medical Inspection Laboratory performed the whole-genome sequencing on the subject's genomic DNA. Using genomic DNA derived from the subject's peripheral blood as the testing material, the DNA was fragmented to prepare a library, and high-throughput sequencing platforms were used for whole-genome sequencing and variant detection. This technical method can detect variations in exonic and intronic regions of genes (including point variations and insertions/deletions within 20 bp), as well as chromosomal aneuploidy variations. It can indicate mitochondrial gene variations, exon-level copy number variations, and large fragment deletions/duplications larger than 30 kb. This method can detect point variations and insertions/deletions within 20 bp in the mitochondrial genome, but cannot detect changes in the copy number of the mitochondrial genome. For mitochondrial variations, combined with the subject's phenotype, only loci included in BGI's internal database with a mutation rate ≥ 10% are reported. This method cannot guarantee the detection of complex genomic structural variations (such as inversions, translocations, and large fragment insertion variations), dynamic variations, low-proportion chimeric variations, and variations located in intergenic regions and gene regulatory regions. This technical method cannot fully cover highly repetitive regions, GC-rich regions, highly complex regions, or pseudogene regions. The quality control indicators for sequencing data are as follows: the average effective sequencing depth of the target region is ≥ 40×, and the proportion of loci with an average depth ≥ 20× is ≥ 90%. The sequencing parameters of the test sample from this patient were as follows: the length of the target region was 2,672,595,047 bp; the coverage of the target region was 99.92%; the average depth of the target region was 59.35×; the proportion of loci in the target region with an average depth >10× was 99.34%; and the proportion of loci in the target region with an average depth >20× was 97.77%. The analysis content mainly includes: the analysis of nuclear genome single nucleotide variations, small fragment insertions/deletions, large fragment copy number variations, loss of heterozygosity variations, structural variations, mitochondrial genome single nucleotide variations and small fragment insertions/deletions, and dynamic repeat expansion variations of specific genes based on next-generation sequencing data. During the detection and analysis process, based on the phenotypic information of the subject provided clinically, the following Human Phenotype Ontology (HPO) and/or Online Mendelian Inheritance in Man (OMIM) terms were used to screen appropriate candidate disease-causing genes for analysis: HP:0001197 (abnormal prenatal development/birth), HP:0001903 (anemia), HP:0001954 (paroxysmal fever), HP:0003319 (cervical spine abnormality), HP:0030431 (osteochondroma), HP:0100636 (paraganglioma), HP:0100711 (thoracic spine abnormality), and HP:0410007 (cartilage abnormality). This test was conducted to analyze the pathogenic genes of monogenic hereditary diseases and mitochondrial genes clearly defined in OMIM (2023Q1) according to the subject's main complaints. Genes related to polygenic susceptibility diseases and complex diseases are not included in this analysis. This test was sponsored by the Project of Enhancing Rare Disease Diagnosis and Treatment Capabilities Supported by the Central Government’s Public Welfare Lottery Fund.

- **Supplementary Table 1.** **The results of blood and urine in bone metabolism and endocrine examinations.**

| **Characteristics** | **Results** | **Reference range** |
| --- | --- | --- |
| **Bone metabolism markers** |  | |
| Alb | 37g/L | 35-52g/L |
| ALP | 91U/L | 45-125U/L |
| Ca | 2.30mmol/L | 2.11-2.52mmol/L |
| P | 1.38mmol/L | 0.85-1.51mmol/L |
| PTH | 13.6pg/mL | 15.0-65.0pg/mL |
| β-CTx | 0.67ng/mL | 0.26-0.51ng/mL |
| T-25OHD | 22.4ng/mL | > 30 ng/mL |
| TP1NP | 62.3ng/mL | / |
| 24hUCa | 4.41mmol/24h | / |
| 24hUP | 19.85mmol/24h | / |
| **Endocrine markers** |  | |
| T4 | 7.3μg/dL | 4.3-12.5μg/dL |
| TSH | 1.59μIU/mL | 0.38-4.34μIU/mL |
| ACTH | 50.3pg/mL | 7.2-63.3 pg/mL |
| GH | 1.0ng/mL | <2.0 ng/mL |
| Total Cortisol | 22.6μg/dL | 4.0-22.3μg/dL |
| NMN | 0.1nmol/L | ≤1.05 nmol/L |
| MN | 0.12nmol/L | ≤0.32 nmol/L |
| 3-MT | 0.011nmol/L | ≤0.036nmol/L |
| 24hUFC | 69.3μg/24h | 13.2-77.2μg/24h |
| 24hE | 4.8μg/24h | ≤15.3μg/24h |
| 24hNE | 19.3μg/24h | ≤106.8μg/24h |
| 24hDA | 307.5μg/24h | ≤546.0μg/24h |

Note: Alb, Albumin; ALP, Alkaline Phosphatase; P, phosphorus; Ca, calcium; PTH, parathyroid hormone; β-CTx, β-isomerized C-terminal telopeptides; T-25OHD, total 25-hydroxyvitamin D; TP1NP, total procollagen type 1 amino- terminal propeptide; T4, thyroxine; TSH, thyroid stimulating hormone; ACTH, adrenocorticotropic hormone; GH, growth hormone; NMN, 3-methoxynorepinephrine; MN, 3-methoxyepinephrine; 3-MT, 3-methoxytyramine; UFC, urinary free cortisol; E, epinephrine; NE, norepinephrine; DA, dopamine. The patients were positioned supine, and blood samples were collected at 8 am. The samples were promptly sent for testing, and some were placed in an ice bath as required.
